# Knowledge and attitudes about influenza and the common cold in Syria post COVID-19: A qualitative study

**DOI:** 10.1016/j.amsu.2022.104166

**Published:** 2022-07-15

**Authors:** Sarya Swed, Hidar Alibrahim, Mhd Amin Alzabibi, Mosa Shibani, Mostafa Hasoon, Haidara Bohsas, Hasan Raslan, Sham Alholiby, Lilas Channiss, Shahm Azzam Alsakka, Rana Alkassab, Salwa Abdulrahman Barou, Aya Kelzia, Hala Al-Abboud, Fatima Naal, Aya Mtanos Jarrous, Nagham Jawish, Schasa Monaf Suliman, Sedra Dashan, Weaam Esmaeel, Baraa Shebli, Weaam Ezzedean, Fateh Kashkash, Abdullah Khouri, Bisher Sawaf, Ameer Kakaje, Ruby M. Kearney, Sherief Ghozy

**Affiliations:** a Faculty of Medicine, Aleppo University, Aleppo, Syria; b Faculty of Medicine, Syrian Private University, Damascus, Syria; c Faculty of Medicine, Damascus University, Damascus, Syria; d Faculty of Medicine, Hama University, Hama, Syria; e Department of Cardiology, Aleppo University Hospital, University of Aleppo, Syria; f Department of Urology, Ibn-Alnafees Hospital, Damascus, Syria; g Department of Pulmonology, Aleppo University Hospital, Aleppo, Syria; h School of Community Health, Charles Sturt University, Albury, Australia; i Neuroradiology Department, Mayo Clinic, Rochester, MN, USA

**Keywords:** Common cold, COVID-19, Influenza, Knowledge, Syria

## Abstract

**Background:**

The common cold and the influenza are common infections that are frequent in the community. In this study, we estimate the level of knowledge regarding those diseases among the Syrian population in the COVID era as it is important to have this knowledge for future health planning and policies.

**Methods:**

A qualitative study was conducted from November to December in 2021. A structured self-administered questionnaire was distributed as Google Forms on social media platforms and hard copies of the questionnaire to patients, their companions, or workers in public hospitals. Chi-square test and Mann Whitney test were used to study the associations between categorical groups.

**Results:**

This study included 13013 participants, 7856 (60.4%) were females, 78.4% were younger than 31 years old, only 3518 (27%) knew that the common cold and the influenza were caused by viruses, 6146 (47.2%) reported that runny nose was the most annoying symptom, 75.6% of the participants believed that antibiotics could kill viruses, and 7674 (58.9%) had fears from symptoms of common cold and influenza because of covid-19. Females were statistically significantly more knowledgeable and had more fears from the infection compared with males.

**Conclusion:**

This study showed a low level of knowledge among the Syrian population. The view of influenza and common cold have changed after COVID as they are now taken more seriously. Many efforts should be made to spread awareness, effective management, and reducing antibiotic misinformation.

## Highlights

• The public in Syria mostly cannot distinguish between common cold and influenza.
• They still believe that antibiotics is the treatment of choice for them.
• COVID-19 has made people more aware and self-conscious of having flu-like symptoms as they can be from having COVID.

## 1 Introduction

The common cold and the influenza are the most common infections in human beings. We usually contract at least one to four infections by one of those diseases annually [1].

The common cold is defined as having less than 10 days of symptoms of acute viral rhinosinusitis, with Rhinoviruses considered the most common cause [2]. Other pathogens can also be Coronaviruses, Adenoviruses and Parainfluenza viruses. In contrast, the influenza is an acute viral respiratory infection caused by negative-strand Ribonucleic acid (RNA). Influenza has three types that infect humans: A, B, and C [3]. The symptoms of the influenza may include cough, diaphoresis, fatigue, headache, myalgia, nasal congestion, rhinorrhea, or sneezing. In contrast, common cold symptoms are typically milder and are not life-threatening [1].

The severity of the symptoms varies according to multiple factors such as the patient's age, long-term medication, chronic conditions, and the efficiency of the immune system [1,4]. Both infections spread through air droplets by coughing, sneezing, and talking (within 6 feet), or any contact with a contaminated surface followed by the touching mucous membranes such as the eyes, nose, or mouth [5].

Today, because of the recurrent mutations in the influenza viruses’ genome, there are no reliable medications to treat these viruses. However, there were multiple studies that suggest a benefit from medication by herbalists to treat and relieve the symptoms of influenza such as Maputo, North American ginseng, Licorice, Berries, Echinacea, Bai Shao pomegranate, and guava tea [6]. Furthermore, some studies revealed that certain medications had an effect in relieving cold symptoms such as paracetamol, chlorphenamine, and phenylephrine [7].

Tonsillitis is considered one of the conditions that is frequently associated with upper respiratory tract infections (URTIs). Tonsillitis may be due to bacterial or viral infection, which is the most common. Viruses that cause tonsillitis are similar to those that cause common cold and influenza [8]. A common pathogen of bacterial tonsillitis is Streptococcus pyogenes [9].

Syria has been in war for 10 years, which has led to deterioration in the standards of living, economy and health conditions, and the spread of poverty and underdevelopment [10]. Furthermore, unawareness prompted many people to randomly take unprescribed antibiotics with respiratory diseases without consulting a specialist doctor in order to avoid additional costs [11]. This is feasible in Syria, because most medications can be taken without a prescription, including antibiotics.

COVID-19 has changed the way people look at any condition with flu-like symptoms, as they can share the same symptoms. Knowing how people perceive respiratory illness during COVID-19 is particularly important as this knowledge can help the system formulate an exit plan, and strategies to open the society to the world that will be acceptable to the society [12]. However, with many perceiving common cold and influenza as COVID symptoms, this would make it more difficult.

Our study was the first to our knowledge that measures the level of awareness among the Syrian population regarding URTIs. This study aimed to assess the public awareness of the common cold and influenza and other variables among the Syrian population to address the misconceptions regarding these diseases, and to study their association with tonsillitis. It also assessed people's perception towards them during COVID-19 era and how it might have changed how they see URTIs symptoms.

## 2 Methods

### 2.1 Study design, setting, and participants

We conducted a qualitative study across Syria, including participants from all the provinces, (Damascus, Rif dimashq, Aleppo, Homs, Tartous, Latakia, Raqqa, Al hasakah, Qamishli, Deir Ezzor, Hama, Idlib, As-Suwayda, and Daraa).

A nationally representative sample of Syrians was included using a structured self-administered questionnaire constructed by two pulmonologists from Aleppo University Hospital and Al-Assad Hospital from Syria. The questionnaire was piloted on 100 persons to ensure its clarity and reliability, with Cronbach's alpha test value of 0.07.

The study was conducted during the period from November 21, 2021 to December 3, 2021. To ensure the correct diversity in the sample and to avoid selection bias, as many residents do not have access to the Internet either because the necessary infrastructure was damaged or because of the poor economic situation, we distributed the questionnaire in two methods: The first method was by posting Google Form on social media platforms (Facebook, Whatsapp, and Twitter). The second method involved administrating paper forms in person to participants and their companions from public places, including hospitals, gardens and universities. These participants were asked to assist by distributing the forms to their families and friends in each of Damascus, Homs, Aleppo, Tartous, Hama, and As-Suwayda governorates. Then, we transferred their answers to Google forms.

The sample size was calculated using raosoft online software available on http://www.raosoft.com/samplesize.html according to data from World Population Prospects [13], the United Nations estimates the Syrian population in 2019 at around 18 million, assuming 1.2% margin of error, 99% confidence level, and 50% response distribution, the recommended sample size was 11512. Admission criteria included Syrian adults over 18 years old. This concluded a sample size of 13013. This research is registered with a unique identifying number of researchregistry7836.

### 2.2 Measures

The questionnaire included general information such as gender, age, social level, ‏financial level, educational level, and whether the participant was a smoker or had a chronic disease. In addition, seven questions were asked about influenza and cold: (1) What is the rate of Infection with influenza or cold in a year? (2) Do you know the differences between influenza and cold?

(3) What is the season that influenza or cold most happens in? (4) Do you feel concerned when a family member has the influenza or cold? (5,6) What is the treatment that you take when you have the influenza and cold in order? (7) Do you think there is a relationship between tonsillitis with influenza or cold?

### 2.3 Statistical analysis

We extracted the data from Google Form directly to an Excel spreadsheet. Then we analyzed the data using Statistical Package for Social Sciences version 25.0 (SPSS Inc., Chicago, IL, United States).

To investigate the general knowledge of both diseases, multiple short statements were assembled describing these diseases; the statements ranged from absolutely wrong statements (e.g., Influenza is a bacterial disease and cold is a viral disease) graded A = −1, “I do not know” statement graded B = 0, partially correct answers (e.g., Both diseases are viruses, but I don't know if the viral cause is the same or different between common cold and influenza) graded C = 1, to complete correct answers (e.g., Both diseases are viruses, but the viruses that cause them are different) graded D = 2.

Fear of infection was assessed through multiple short statements as the following: A = 0 (I never feel afraid), A = 1 (I feel a little afraid), A = 2 (Yes, I feel afraid for one of the reasons: Suspicion of covid-19 infection, respiratory diseases that weaken immunity, when symptoms are severe). To study the association between the fear of infection from family members, and participants’ basic characteristics, Chi-square test was used with an adopted level of significance of 5%.

The infection rate per year was also assessed through multiple short answers. The first group IR = 1 had a variable infection rate from one year to the other, the second group IR = 2 had an infection rate of three times or less per year, and finally, the third group IR = 3 had an infection rate of more than three times per year.

To study the association of the awareness of the difference between the two diseases and the gender, a Mann-Whitney test was applied.

### 2.4 Ethical consideration

Ethical approval was obtained from the Scientific Research Ethics Committee of Damascus and Aleppo Universities. The participation was voluntary, and online/written informed consent was obtained from all participants. The privacy and confidentiality of the collected data have also been maintained.

## 3 Results

Following the application of eligibility criteria, 13013 participants were enrolled in the study. Overall, 7407 (56.9%) filled in the paper forms. A total of 7856 (60.4%) participants were females, while males comprised 39.6% of participants. Various age groups participated in this study; the group age 21–25 years old was the most dominant group in the study comprising 42.2% of all study population. In general, the study included mainly youth, as 78.4% of the total population were younger than 31 years old. The participants were on variable levels of education; however, university students were the dominant part of the sample, comprising 80.1% of all participants, and around 25.2% of participants were active smokers. Further information regarding the basic characteristics of the population can be found in (Table 1).

**Table 1:** Basic characteristics of the study population.

Variable	Number(%)
Age	
18–20	3348(25.7%)
21–25	5492 (42.2%)
26–30	1361(10.5%)
31–40	1292(9.9%)
41–50	884(6.8%)
51–80	636(4.9%)
Gender	
Male	5157 (39.6%)
Female	7856 (60.4%)
Economical level	
Low	1334(10.3%)
Middle	8163(62.7%)
Good	3516(27.0%
Social status	
Single	8786(67.5%)
In a relation	734(5.6%)
Married	3145(24.2%)
Divorced	187(1.4%)
Widowed	161(1.2%)
Educational level	
Medical student	4429(34.0%)
Non-medical student	6001(46.1%)
Reached the primary stage of school	1115(8.6%)
Reached the secondary stage of school	633(4.9%)
Illiterate	180(1.4%)
Master	524(4.0%)
PhD	131(1.0%)
Smoking	
No	9730(74.8%)
Yes	3283(25.2%)
1-5 cigarette per day	764(5.9%)
6-10 cigarette per day	502(3.9%)
11-15 cigarette per day	419(3.2%)
16-20 cigarette per day	793(6.1%)
21-25 cigarette per day	291(2.2%)
Above 26 cigarette per day	428(3.3%)
Chronic disease	
No	11706(89.9%)
Yes	1307(10.1%)

**Fig. 1:**
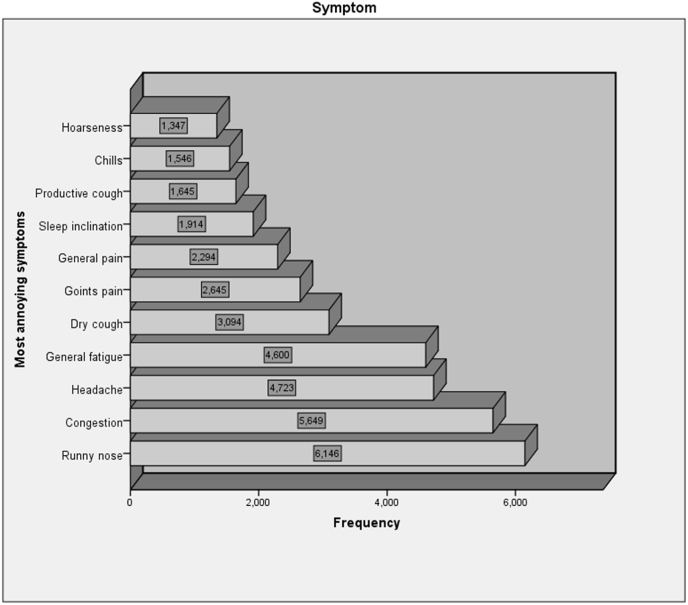
Annoying reported symptoms.

The most annoying reported symptom was “runny nose”, reported by 6146 (47.2%) of the participants, followed by congestion, reported by 5649 (43.4%) of the participants. The least annoying reported symptoms were hoarseness and chills, reported by 1347 (43.4%), and 1546 (11.8%) of the participants, respectively (Fig. 1).

**Fig. 2:**
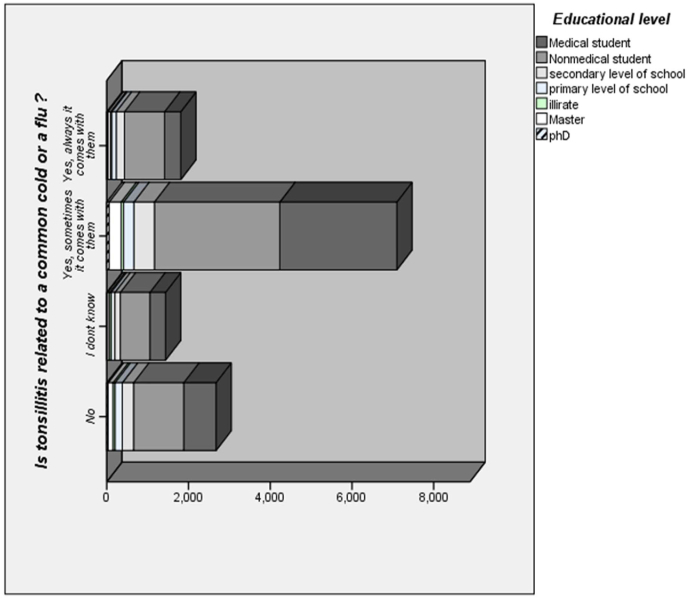
Is tonsillitis related to the common cold or flu?.

The most common answer for the question about the association between tonsilitis and common cold and influenza was “yes, sometimes it comes with them” reported by 7000 (53.7%) participants, followed by the answer “No” 2731 (20.9%) who stated there was no association between the two diseases (Fig. 2).

“Influenza has more severe symptoms or symptoms of influenza include the entire body and symptoms of a common cold include the respiratory system only” was the most common answer reported by the participants 4880 (37.5%), followed by “I do not know” reported by 2467 (19%) of the total population. (Table 2).

**Table 2:** Knowledge regarding the common cold and flu.

	Number	Percentage(%)
Flu has more severe symptoms or symptoms of flu include the entire body and symptoms of a common cold include the respiratory system only	4880	37.5%
Both diseases are viruses, but I don't know if the viral cause is the same or different between the common cold and flu	1555	11.9%
Both diseases are viruses, but the viruses that cause them differ	1963	15.1%
I don't know	2467	**19.0%**
The common cold is a bacterial disease, and the flu is a viral disease	1136	**8.7%**
Flu is a bacterial disease and cold is a viral disease	501	**3.8%**
Both are related to COVID-19	298	**2.3%**
Common cold has more severe symptoms or symptoms of the common cold include the entire body and symptoms of the flu include the respiratory system only	213	**1.6%**

**Fig. 3:**
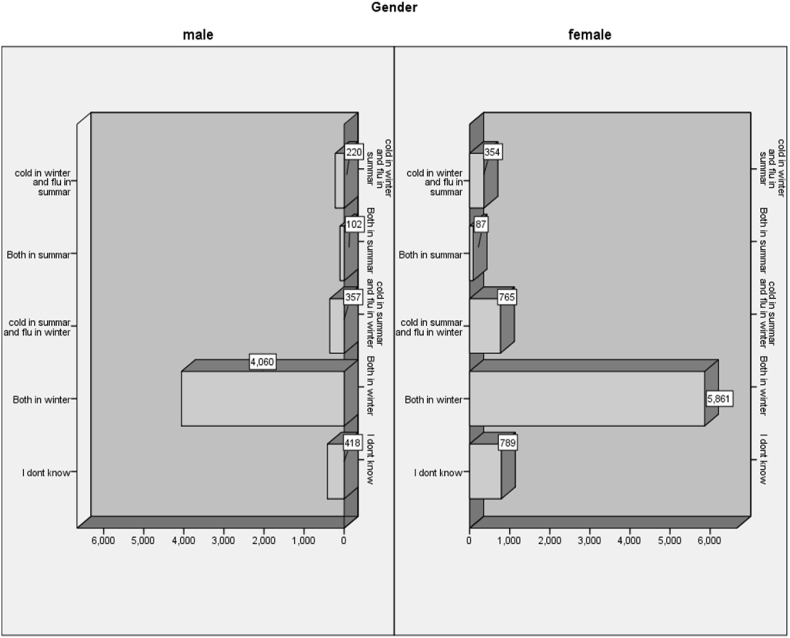
Knowledge of males and females regarding the timing of both common cold and flu.

Comparing the knowledge of males and females regarding the timing of both diseases, the two groups were almost identical, with the most common answer being “Both in winter”, reported by 5861 out of 7856 females, and 4060 out of 5157 males (Fig. 3).

**Fig. 4:**
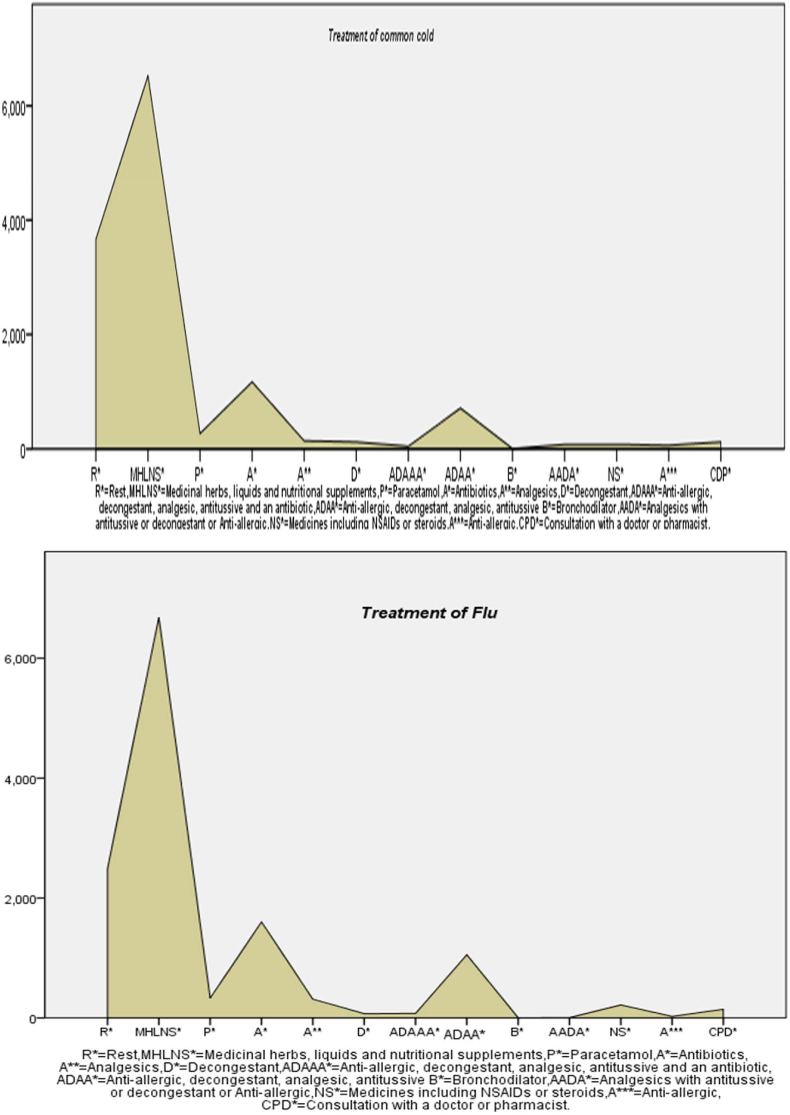
Knowledge regarding the management of the common cold and flu.

During the estimation of the awareness of the proper management of these two conditions, the first two most common management options reported were “Medicinal herbs, Liquids and Nutritional Supplements (MHLNS)” and “Rest” for both diseases. Interestingly, the third most common management option reported by the population was “Antibiotics'' despite being viral diseases (Fig. 4).

A statistically significant association was found between the gender of the participant and the fear of infection (p < 0.001) with the females having more fear in this regard (33.5% from the total population were females in the group A = 2). In addition, the association between the economic level and fear of infection was also statistically significant (p < 0.001) (Table 3).

**Table 3:** The association between participants’ characteristics and the fear of infection.

Variables	A = 0(%) n = 3638 n(%) or median (Q1-Q3)	A = 1(%) n = 2301 n(%) or median (Q1-Q3)	A = 2(%) n = 7674 n(%) or median (Q1-Q3)	X^2^	P-value
Gender				23.85	<0.001
Male	1564(12.0%)	881(6.8%)	2712(20.8%)		
Female	2074(15.9%)	1420(10.9%)	4362(33.5%)		
Age				10.50	0.39
18–20	936(7.2%)	565(4.3%)	1847(14.2%)		
21–25	1565(12.0%)	960(7.4%)	2967(22.8%)		
26–30	364(2.8%)	244(1.9%)	753(5.8%)		
31–40	339(2.6%)	261(2.0%)	692(5.3%)		
41–50	248(1.9%)	155(1.2%)	481(3.7%)		
51–80	186(1.4%)	116(0.9%)	334(2.6%)		
Economical level				35.36	**<0.001**
Low	402(3.1%)	241(1.9%)	347(2.7%)		
Middle	2214(17.0%)	1497(11.5%)	2535(19.5%)		
Good	1022(7.9%)	583(4.3%)	1032(7.9%)		
Social level				0.034	0.062
Single	2487(19.1%)	1502(11.5%)	4797(36.9%)		
In relation	186(1.4%)	137(1.1%)	411(3.2%)		
Married	879(6.8%)	596(4.6%)	1670(12.8%)		
Divorced	50(0.4%)	41(0.3%)	96(0.7%)		
Widowed	36(0.3%)	25(0.2%)	100(0.8%)		
Educational level				0.027	0.635
M. student^a^	1249(9.6%)	757(5.8%)	2423(18.6%)		
NM. Student^b^	1692(13.0%)	1048(8.1%)	3261(25.1%)		
Primary stage^c^	177(1.4%)	118(0.9%)	338(2.6%)		
Secondary stage^d^	308(2.4%)	203(1.6%)	604(4.6%)		
Illiterate	46(0.4%)	41(0.3%)	93(0.7%)		
Master	132(1.0%)	108(0.8%)	284(2.2%)		
PhD	34(0.3%)	26(0.2%)	71(0.5%)		
Smoking				5.597	0.61
No	2670(20.5%)	1719(13.2%)	5340(41.0%)		
Yes	968(7.4%)	582(4.5%)	1734(13.3%)		
Chronic disease				1.39	0.50
No	3288(25.3%)	2059(15.8%)	6355(48.8%)		
Yes	350(2.7%)	242(1.9%)	719(5.5%)		

a Medical student.

b Nonmedical student.

c Reached the primary stage of school.

d Reached the secondary stage of school.

Most participants fell into the second group (IR = 2) with a total number of 6258 out of 13013 participants, followed by the third group IR = 3 (4175 out 13013 participants). A statistically significant association was found between the infection rate and all the variables of the participants’ basic characteristics (p = 0.001 for all tests except for the association with chronic disease p = 0.017) (Table 4).

**Table 4:** The association between participants’ characteristics and the infection rate.

Variables	IR = −1(%) n = 2580 n(%) or median (Q1-Q3)	IR = 2(%) n = 6258 n(%) or median (Q1-Q3)	IR = 3 (%) n = 4175 n(%) or median (Q1-Q3)	X^2^	P-value
Gender				16.53	<0.001
Male	1247(9.6%)	3362(25.8%)	548(4.2%)		
Female	1333(10.2%)	2896(22.3%)	3627(27.9%)		
Age				256.11	<0.001
18–20	713(26.6%)	1609(2.0%)	1026(3.9%)		
21–25	1221(15.2%)	2527(6.4%)	1744(6.5%)		
26–30	255(3.4%)	635(1.4%)	471(1.9%)		
31–40	190(3.7%)	649(1,1%)	453(1.3%)		
41–50	125(2.9%)	463(0.6%)	296(0.8%)		
51–80	76(2.3%)	375(0.5%)	185(0.5%)		
Economical level				60.44	<0.001
Low	253(1.9%)	749(5.8%)	332(2.6%)		
Middle	1543(11.9%)	3946(30.3%)	2674(20.5%)		
Good	784(6.0%)	1563(12.0%)	1169(9.0%)		
Social level				93.41	<0.001
Single	1908(14.7%)	4187(32.2%)	2691(20.7%)		
In relation	161(1.2%)	342(2.6%)	231(1.8%)		
Married	455(3.5%)	1562(12.0%)	1128(8.7%)		
Divorced	33(0.3%)	81(0.6%)	73(0.6%)		
Widowed	23(0.2%)	86(0.7%)	52(0.4%)		
Educational level				765.11	<0.001
M. student*	1132(11.8%)	1890(14.5%)	1407(10.8%)		
NM. Student**	1012(7.8%)	3098(23.8%)	1891(14.5%)		
Primary stage^a^	80(0.6%)	343(2.6%)	210(1.6%)		
Secondary stage^b^	174(1.3%)	533(4.1%)	408(3.1%)		
Illiterate	23(0.2%)	104(0.8%)	53(0.4%)		
Master	136(1.0%)	214(1.6%)	174(1.3%)		
PhD	23(0.2%)	76(0.6%)	32(0.2%)		
Smoking				45.50	<0.001
No	1928(14.8%)	4309(33.1%)	3492(26.8%)		
Yes	652(5.0%)	1949(15.0%)	683(5.2%)		
Chronic disease				2.72	0.017
No	2311(17.8%)	5591(43.0%)	3800(29.2%)		
Yes	269(2.1%)	667(5.1%)	375(2.9%)		

**& Cigarette per day**.

**#Pearson Chi-Square(Smoking)**.

a **Reached the primary stage of school**.

b **Reached the secondary stage of school**.

Mann Whitney test showed a statistically significant difference between females and males (p = 0.043), with females being more aware of the difference between the two diseases (Table 5).

**Table 5:** Assessing the association between the Awareness of difference between common cold and flu and gender.

	All	Male	Female	p-value
Awareness of difference between common cold and flu	2(1.0–2.0)	1(1.0–1.0)	2(2.0–2.0)	0.034*


Mann Whitney (Gender – difference between common cold and flu).

## 4 Discussion

To our best knowledge, this is the first nationally representative study in Syria to study the population perception about influenza and the common cold. Our population consists mainly of young adults. This is somewhat expected since Syrian society is considered a young society. According to the latest data of the Central Bureau of Statistics in Syria, almost half of the Syrian population (40%) was under 24 years old around the time of this study, and (25.5%) of them were 25–44 years old [14].

Knowing the causative agents of the common cold and influenza is essential in understanding the nature of the disease and its proper management. Our results found that 3518 (27%) knew that the common cold and influenza were caused by viruses, but only 1963 (15.1%) knew that they were caused by different viruses. A higher level of knowledge was found in a UK study, which found that most of the British people answered that ‘the air’ and viruses were the causes of upper respiratory diseases, and only 13% of their respondents believed that the cold and influenza were the same [15]. This may be due to the Syrian war being ongoing for more than a decade, which affected all educational and awareness events in the country.

As most influenza and flu cases are only managed symptomatically, complementary and alternative therapies for colds and influenza such as herbs were commonly used. Many participants used herbal medicines, hot liquids, and nutritional supplements which showed good efficacy in reducing the length and severity of colds [6]. Unfortunately, antibiotics were the third most common management option reported in our study despite being ineffective. A previous study on the Syrian population found that 75.6% of the participants believed that antibiotics can kill viruses [11]. Other studies in Saudi Arabia and the UK found a similarly high misconception with 66% and 44% of their population believing that antibiotics could cure the common cold, respectively. Another study in Manhattan, USA reported that 88% of the enrolled population agreed that bacteria cause the common cold and influenza [16]. Moreover, another Syrian study documented that at least (36%) of the participants expected their doctor to prescribe antibiotics every time they experienced symptoms of the common cold [11], which subsequently led to prescribing antibiotics for viral infections due to the pressure they felt from patients [17].

Runny nose, congestion, cough, and sneezing are considered some of the most common symptoms of both diseases [1]. However, the manifestations can vary between individuals and are influenced by several factors such as the type of the pathogens, age, comorbidities, and the host immunological status. Our participants considered runny nose and congestion to be the most annoying symptoms they experienced during the illness.

Most cases of tonsillitis are caused by a viral infection like rhinoviruses, and the influenza virus, the same viruses that cause common cold and influenza. Most of the population seems to be aware of the association between these illnesses [9]. Since the influenza and the common cold have some comparable symptoms, it can be difficult for the public to distinguish between them [1], which was similar to our findings.

Suspicion of covid-19 infection led to 7674 (58.9%) participants to have fears from the symptoms of common cold and influenza, as they may share common symptoms. A previous study in the Syrian population found that 61.6% thought coronavirus posed a major risk to people in Syria [18]. A study regarding European countries' populations found that most participants perceived a common cold as a harmless or mild disease [19]. This showed the effect of covid-19 on URTIs perception among our sample and the public in general. This fear of common cold and influenza symptoms was more prominent among middle class 2535 (19.5%) in comparison to 1032 (7.9%) of the higher-class people. This might be due to lower standard of living and quality of life. Knowing what people perceive COVID and other respiratory illnesses might help to formulate policies and strategies to enable more evidence-based approach to de-escalate restrictions along with other papers [20].

A peak in cases has been reported during the winter for a wide range of URTIs caused by different viruses belonging to different families [21]. This is due to several factors related to both the viruses and the host [3,21]. It appears that most of our participants are familiar with this fact. This can be explained by the fact that most people who contracted influenza or common cold in the past, had it in the colder months of the year, so people would associate their infection with the time of the year they had it.

Our results demonstrated that females are more aware of the difference between the two diseases. Several previous studies were consistent about females being more health conscious compared to males [22,23]. Furthermore, a study by Bidmon et al. suggested that females had more social motives to find health-related information on the internet and enjoy the searching process more than men, and they may be more influenced by health-related awareness campaigns [23]. This paper followed strengthening the reporting of cohort, cross-sectional and case-control studies (STROCSS) to ensure good quality reporting observation studies [24].

A low level of knowledge could negatively affect the country's resources and patients' quality of life. A general awareness program targeting healthcare professionals and the public is highly recommended to help increase knowledge and improve public attitude towards treatment and effective management and correcting the misunderstandings regarding antibiotics use. Furthermore, long-term prospective studies should be conducted to address the patterns of treatment, financial burden, and productivity loss among patients. This awareness is particularly necessary when new epidemics rise. This was particularly prominent with COVID-19, when despite containment and quarantine efforts, case numbers continued to rise [25]. It also led to a further deterioration of the socioeconomic status which added even more to the burden in Syria [26] due to more than 90% being under poverty line. Furthermore, the chaos that was prevalent at the beginning of COVID-19 was so overwhelming, that we can see how many medications were experimentally used or were later found to be ineffective and the guidelines would be frequently updated [27]. This research was reported according to qualitative research criteria [28].

## 5 Conclusions

The Syrian public awareness about the common cold and influenza causative agent, and management is relatively low. Most people's fear of flu-like symptoms were because they had fears of acquiring COVID-19. A good proportion of people were expecting antibiotics on every occasion that they had the flu, and did not know that viral illnesses are not treated with antibiotics. Decision-makers in the Syrian Ministry of Health must consider this issue seriously and address it.

## Limitation

Selection bias was minimized by distributing the questionnaire online and in person, to get a representative sample of people with low economic status, since the online questionnaire may be filled mostly by young age, good economic status groups. Self-reporting led to Recall bias.

Very few papers in the medical literature discuss the same subject, which made it difficult to compare our results with others.

## Availability of data and materials

The datasets used and/or analyzed during the current study are made available by the corresponding author Ameer Kakaje on a reasonable request.

## Provenance and peer review

Not commissioned, externally peer-reviewed.

## Sources of funding

No funding

## Ethical approval

Ethical approval was obtained from the Scientific Research Ethics Committee of Damascus and Aleppo Universities, the participation was voluntary, and written informed consent was obtained from all participants. The privacy and confidentiality of the collected data have also been maintained.

## Consent

The Research Ethics Committee in the Syrian Private University, Damascus and Aleppo Universities, and the ethical committees in the concerned hospitals approved the study protocol. Written informed consent was obtained from every participant prior to participation. All procedures performed in studies involving human participants were in accordance with the ethical standards of the institutional and/or national research committee and with the 1964 Helsinki declaration and its later amendments or comparable ethical standards.

## Author contribution

SS (first author + guarantor), HA, MAA, MHA, MS, MH, HB, HR, SA, LC, SAA, RA, SAB and MAK conceptualized the study, participated in the design, wrote the study protocol, did a literature search, and drafted the manuscript. SS performed the statistical analysis and did a literature search. AK (corresponding + second author), HSG, KRM, AK, HAA, FN, AMJ, NJ, SMS, SD, WE, BS, NS, WE, FK, AK, SA, BS, MYE, RMK and SG (senior author) participated in the design, did a literature search and revision of the draft. All authors read and approved the final draft.

## Registration of research studies

1. Name of the registry: Research Registry
2. Unique Identifying number or registration ID: researchregistry7836
3. Hyperlink to your specific registration (must be publicly accessible and will be checked): https://www.researchregistry.com/browse-the-registry#home/registrationdetails/6265de96733854001e8d9c1c/

## Guarantor

Sarya Swed is the guarantor.

## Declaration of competing interest

No conflict of interest.

## List of abbreviations

RNA: Ribonucleic acid
URTIs: upper respiratory tract infections
SPSS: Social Sciences version
MHLNS: Medicinal herbs, Liquids and Nutritional Supplements
